# Supplementary material for: Hippocampal Transcriptome Analysis in a Mouse Model of Chronic Unpredictable Stress Insomnia
Source: Biomedicines. 2025 May 15;13(5):1205. doi: 10.3390/biomedicines13051205 (PMC12108738; doi:10.3390/biomedicines13051205)
Supplement: Supplementary file 1 [file biomedicines-13-01205-s001.zip › biomedicines-3521469-supplementary.pdf]

Table S1 Open-field test data (Mean±SD)

Group	TDMC (%)	Time spnet in the center (s)	TDMP (%)	time spent in the periphery (s)
Control	9.77±2.57	38.42±12.69	54.49±7.00	217.01±21.42
CUMS	6.68±2.91*	24.00±8.20**	63.33±10.28*	239.01±18.69*
CUMS+Noise	7.60±3.72	22.85±7.72##	62.07±9.10	237.12±21.49#
P(CUMS VS Control)	0.045	0.005	0.046	0.033
P(CUMSN VS Control)	0.152	0.003	0.083	0.049
F	2.350	7.067	2.601	3.160
df	(2.24)	(2.24)	(2.24)	(2.24)
P(between-gro up variation)	0.117	0.004	0.095	0.060

Note:In the open-field test (ANOVA, $n = 9$ per group), the observation indicators include the percentage of total distance moved in the center (TDMC%) , time spent in the center (TPC), percentage of total distance moved in the periphery (TDMP%), and time spent in the periphery (TPP). *$p < 0.05$ **$p < 0.01$, CUMS group compared to control group. #$p < 0.05$ ##$p < 0.01$, CUMS+Noise group compared to control group.

Table S2 Elevated plus maze test data (Mean±SD)

Group	Percentage of open arm time(%)	Percentage of entries to the open arms (%)	Total distance moved (cm)
Control	10.88±3.99	37.41±9.83	9.12±3.70
CUMS	7.33±3.51*	28.53±8.44	3.79±1.82***
CUMS+Noise	2.40±0.86###	25.15±9.37##	2.48±1.74###
P(CUMS VS Control)	0.052	0.026	0.0002
P(CUMSN VS Control)	0.00952	0.000008	0.000013
F	4.237	16.487	16.65
df	(2.24)	(2.24)	(2.24)
P(between-gro up variation)	0.027	0.000036	0.000029

Note:In the elevated plus maze test (ANOVA, $n = 9$ per group), the observation

indicators include the percentage of time spent in the open arms (OAT%), the percentage of entries to the open arms (OAE%), and the percentage of total distance moved to the open arms (OATDM%). #$p < 0.05$ ##$p < 0.01$ ###$p <$ 0.001,CUMS+Noise group compared to control group.

Table S3 Cort concentration (Mean±SD)

Group	Concentration (ng/mL)
Control	24.98±7.14
CUMS	42.08±9.38*
CUMS+Noise	49.12±17.69##
P(CUMS VS Control)	0.048
P(CUMSN VS Control)	0.009
F	5.115
df	(2.12)
P(between-group variation)	0.025

Note:Corticosterone concentrations (ANOVA, $n = 5$ per group). *$p < 0.05$, CUMS group compared to control group. ##$p < 0.01$, CUMS+Noise group compared to control group.

Table S4 Pentobarbital sodium sleep test data (Mean±SD)

Group	Sleep Latency (min)	Sleep Duration (min)
Control	7.25±2.31	57.88±7.43
CUMS	4.38±1.06	59.63±10.45
CUMS+Noise	8.75±4.83	40.38±15.95##
P(CUMS VS Control)	0.083	0.770
P(CUMSN VS Control)	0.352	0.007
F	3.975	6.491
df	(2.21)	(2.21)
P(between-group variation)	0.034	0.006

Note:In the pentobarbital sodium-induced sleep test, sleep duration (ANOVA, $n = 8$

per group) and sleep latency (ANOVA, $n = 8$ per group) were recorded. ##$p < 0.01$, CUMS+Noise group compared to control group.

Table S5 Percentage of dark phase sleep data ($n = 5$)

Group	Percentage of dark phase sleep (%)
Control	60.34±8.60
CUMS	55.15±4.21
CUMS+Noise	51.43±5.06#
P(CUMS VS Control)	0.213
P(CUMSN VS Control)	0.044
F	2.566
df	(2.12)
P(between-group variation)	0.118

Note:Sleep percentage in the dark phase (ANOVA, $n = 5$ per group). #$p < 0.05$, CUMS+Noise group compared to control group.

Table S6 Multiple sleep time intervals data ($n = 5$)

Group	20-21 time interval	22-23 time interval	27-28 time interval	29-30 time interval	31-32 time interval
P(CUMS VS Control)	0.539	1.000	0.980	0.688	0.433
P(CUMSN VS Control)	0.042	0.025	0.032	0.027	0.028
df	2	2	2	2	2
Chi-Square	6.067	7.385	6.615	6.680	6.772

Note: Kruskal-Wallis was used to analyze the sleep recordings and scoring. There was a significant difference in the CUMS+Noise group compared to the control group, $p < 0.05$.

Table S7 Single sleep duration data ($n = 5$)

Group	120s	240s	480s	1920s
P(CUMS VS Control)	1.000	0.980	1.000	0.172
P(CUMSN VS Control)	0.049	0.032	0.043	0.037
df	2	2	2	2
Chi-Square	6.020	6.615	6.731	6.580

Note: Kruskal-Wallis was used to analyze the sleep recordings and scoring. There was a significant difference in the CUMS+Noise group compared to the control group, $p < 0.05$.

Table S8 Module-trait relationships data (*n* = 5)

R/Pvalue	Cort	SD	TPC	OAT%	OAE%
MEred	-0.218/0.546	0.281/0.431	-0.033/0.928	-0.029/0.937	-0.656/0.039
MEpurple	0.421/0.225	-0.126/0.729	-0.115/0.752	-0.157/0.665	-0.485/0.155
MEturquoise	-0.055/0.880	-0.206/0.569	0.166/0.647	-0.058/0.874	0.238/0.508
MEyellow	-0.441/0.202	0.394/0.260	0.270/0.451	0.466/0.174	0.116/0.749
MEblue	-0.182/0.615	-0.241/0.501	-0.418/0.229	-0.277/0.439	-0.243/0.499
MEgreen	-0.583/0.077	0.712/0.021	0.286/0.423	0.397/0.256	0.119/0.744
MEblack	0.125/0.731	0.045/0.901	-0.201/0.578	0.073/0.841	-0.124/0.732
MEmagenta	0.427/0.218	-0.566/0.088	-0.408/0.241	-0.245/0.494	-0.300/0.400
MEpink	-0.088/0.808	0.044/0.903	0.042/0.908	0.088/0.809	0.532/0.113
MEbrown	-0.555/0.096	0.753/0.012	0.831/0.003	0.650/0.042	0.825/0.003
MEgreenyellow	-0.072/0.843	0.205/0.570	0.345/0.329	0.053/0.884	-0.113/0.757
MEgrey	0.190/0.598	0.028/0.939	-0.030/0.935	-0.163/0.652	-0.128/0.725

Note: Significant differences were considered when R > 0.6 or R < -0.6 and $p < 0.05$. Cort: corticosterone concentrations, SD: sleep duration,TPC: time spnet in the center,

OAE%: percentage of entries to the open arms, OAT%: percentage of time spent in the open arms.

Table S9 KEGG Pathway

Description	Gene ratio	Pvalue
Glutamatergic synapse	2.791341112	1.29E-07
Aldosterone synthesis and secretion	2.680052349	2.05E-06
MAPK signaling pathway	1.85962816	6.84E-06
Growth hormone synthesis, secretion and action	2.44723613	8.44E-06
Neuroactive ligand-receptor interaction	1.716023953	1.13E-05
Calcium signaling pathway	1.883767564	3.10E-05
Salivary secretion	2.5975892	3.25E-05
Oxytocin signaling pathway	2.130298021	3.65E-05
Thyroid hormone synthesis	2.699553769	4.32E-05
Retrograde endocannabinoid signaling	2.13122666	4.81E-05
Glycosphingolipid biosynthesis - lacto and neolacto series	4.04386597	7.39E-05
Axon guidance	1.975015203	7.89E-05
Relaxin signaling pathway	2.11911116	0.000166286
Circadian entrainment	2.25301104	0.000287921
Oocyte meiosis	2.085431707	0.000375892
cGMP-PKG signaling pathway	1.884020793	0.000384955
Apoptosis	2.010039262	0.00039981
Apoptosis - multiple species	3.285641101	0.000529375
GABAergic synapse	2.244572797	0.000579837
TNF signaling pathway	2.046983482	0.000844462

Note:Significant differences were considered when $p < 0.05$.

Table S10 GO Pathway

Biological Processes

Description	Gene ratio	Pvalue
chemical synaptic transmission	38/2172	1.71157E-09
ion transmembrane transport	60/2172	2.92069E-09
intracellular signal transduction	65/2172	8.25289E-09
nervous system development	70/2172	2.02214E-08
positive regulation of synapse assembly	21/2172	6.26627E-07
axon guidance	37/2172	1.04248E-06
protein glycosylation	22/2172	1.43838E-06
positive regulation of neuron projection	35/2172	4.00559E-06

development		
positive regulation of protein phosphorylation	44/2172	5.3033E-06
rhythmic process	30/2172	7.14731E-06
neuron maturation	9/2172	8.01824E-06
heterophilic cell-cell adhesion via plasma membrane cell adhesion molecules	15/2172	8.22074E-06
regulation of membrane potential	23/2172	1.00643E-05
positive regulation of synaptic transmission, GABAergic	9/2172	1.35003E-05
synaptic membrane adhesion	11/2172	1.58513E-05
negative regulation of cell proliferation	57/2172	1.73987E-05
homophilic cell adhesion via plasma membrane adhesion molecules	23/2172	2.28011E-05
synaptic vesicle endocytosis	14/2172	2.33891E-05
oligosaccharide biosynthetic process	8/2172	2.77391E-05
synapse assembly	15/2172	2.91971E-05

Note:Significant differences were considered when $p < 0.05$.

Molecular Functions

Description	Gene ratio	Pvalue
ion channel activity	43/2172	3.90594E-11
ligand-gated ion channel activity involved in regulation of presynaptic membrane potential	12/2172	1.11554E-07
transmitter-gated ion channel activity involved in regulation of postsynaptic membrane potential	17/2172	1.42758E-07
transferase activity, transferring glycosyl groups	41/2172	4.32234E-07
phosphorus-oxygen lyase activity	10/2172	1.31798E-06
PDZ domain binding	27/2172	2.51056E-06
transcription coactivator binding	15/2172	2.29911E-05
protein serine/threonine kinase activity	59/2172	4.43072E-05
ionotropic glutamate receptor binding	14/2172	4.94391E-05
extracellular ligand-gated ion channel activity	12/2172	4.95675E-05
fucosyltransferase activity	6/2172	5.1515E-05
adenylate cyclase activity	8/2172	7.41142E-05
rRNA binding	13/2172	8.37969E-05
phosphoprotein phosphatase activity	26/2172	9.41673E-05
phosphatase activity	25/2172	0.000104718

phosphatidylinositol-3-phosphatase activity	6/2172	0.000195787
ubiquitin protein ligase activity	48/2172	0.000216347
microtubule binding	39/2172	0.000256723
ionotropic glutamate receptor activity	7/2172	0.000381682
cadherin binding	15/2172	0.000388654

Note:Significant differences were considered when $p < 0.05$.

Cellular Components

Description	Gene ratio	Pvalue
postsynaptic membrane	70/2172	1.88983E-16
axon	90/2172	1.13557E-14
presynaptic membrane	48/2172	6.99202E-14
postsynaptic density membrane	35/2172	6.12122E-13
neuron projection	73/2172	1.73795E-12
GABA-ergic synapse	34/2172	3.40325E-09
Schaffer collateral - CA1 synapse	33/2172	6.86811E-08
synaptic vesicle membrane	31/2172	7.79392E-08
presynaptic active zone membrane	20/2172	9.42349E-08
postsynaptic density	51/2172	1.33707E-07
presynapse	42/2172	5.05433E-07
synaptic vesicle	34/2172	2.29091E-06
dendritic spine	35/2172	3.53052E-06
somatodendritic compartment	10/2172	3.94895E-06
excitatory synapse	15/2172	4.68279E-06
growth cone	31/2172	9.78849E-06
postsynaptic specialization membrane	13/2172	2.25346E-05
dendritic shaft	17/2172	4.53482E-05
synaptic membrane	17/2172	5.50459E-05
ion channel complex	26/2172	6.48694E-05

Note:Significant differences were considered when $p < 0.05$.

Table S11 Module-trait relationships data ($n = 5$)

Sample	Cort	SD	TPC	OAT	OAE	Alb	Npsr1	P2rx1
Cort	1/0	-0.527/0.118	-0.524/0.120	-0.598/0.068	-0.162/0.656	0.289/0.417	-0.409/0.241	-0.448/0.194
SD	-0.527/0.118	1/0	0.782/0.007	0.741/0.014	0.471/0.169	-0.717/0.019	0.633/0.049	0.797/0.006
TPC	-0.524/0.120	0.782/0.007	1/0	0.888/0.001	0.717/0.020	-0.792/0.006	0.727/0.017	0.796/0.006

OAT	-0.598/0.068	0.741/0.014	0.888/0.001	1/0	0.640/0.046	-0.606/0.063	0.529/0.116	0.589/0.073
OAE	-0.162/0.656	0.471/0.169	0.717/0.020	0.640/0.046	1/0	-0.463/0.177	0.638/0.047	0.633/0.049
Alb	0.289/0.417	-0.717/0.019	-0.792/0.006	-0.606/0.063	-0.463/0.177	1/0	-0.474/0.167	-0.739/0.015
Npsr1	-0.409/0.241	0.633/0.049	0.727/0.017	0.529/0.116	0.638/0.047	-0.473/0.167	1/0	0.636/0.048
P2rx1	-0.448/0.194	0.797/0.006	0.796/0.006	0.589/0.073	0.633/0.049	-0.739/0.015	0.636/0.048	1/0

Note: Significant differences were considered when $R > 0.6$ or $R < -0.6$ and $P < 0.05$. Cort: corticosterone concentrations, SD: sleep duration,TPC: time spnet in the center, OAE%: percentage of entries to the open arms, OAT%: percentage of time spent in the open arms.

Table S12 Hub genes RT-PCR data (Mean±SD)

Group	Alb	Npsr1	P2rx1
Control	0.746±0.209	1.649±0.780	0.980±0.333
CUMS+Noise	1.558±0.445#	1.182±0.426	0.958±0.278
P(CUMSN VS Control)	0.016	0.334	0.790
df	6	6	6
t	-3.305	1.051	0.102
F	2.566	0.670	0.077
P	0.172	0.444	0.790

Note: qRT-PCR validation results (Student's *t*-test, $n = 4$ per group); the relative expression of each gene was calculated using the comparative Ct method ($2^{-\Delta\Delta Ct}$). #$p < 0.05$, CUMS+Noise group compared to control group.
